# Prevalence and risk factors of intimate partner violence among women in four districts of the central region of Ghana: Baseline findings from a cluster randomised controlled trial

**DOI:** 10.1371/journal.pone.0200874

**Published:** 2018-07-19

**Authors:** Deda Ogum Alangea, Adolphina Addoley Addo-Lartey, Yandisa Sikweyiya, Esnat Dorothy Chirwa, Dorcas Coker-Appiah, Rachel Jewkes, Richard Mawuena Kofi Adanu

**Affiliations:** 1 Department of Population, Family and Reproductive Health, School of Public Health, University of Ghana, Accra, Ghana; 2 Department of Epidemiology and Disease Control, School of Public Health, University of Ghana, Accra, Ghana; 3 Gender and Health Research Unit, South African Medical Research Council, Pretoria, South Africa; 4 Gender Studies and Human Rights Documentation Centre, Accra, Ghana; Erasmus Medical Center, NETHERLANDS

## Abstract

Intimate partner violence (IPV) is a significant global public health problem. Understanding risk factors is crucial for developing prevention programmes. Yet, little evidence exists on population-based prevalence and risk factors for IPV in West Africa. Our objective was to measure both lifetime and past year prevalence of IPV and to determine factors associated with past year physical or sexual IPV experience. This population-based survey involved 2000 randomly selected women aged 18 to 49 years living in 40 localities within four districts of the Central Region of Ghana. Questionnaires were interviewer-administered from February to May 2016. Respondents were currently or ever-partnered, and resident in study area ≥12months preceding the survey. Data collected included: socio-demographics; sexual behavior; mental health and substance use; employment status; 12-month and lifetime experience of violence; household food insecurity; gender norms/attitudes; partner characteristics and childhood trauma. Logistic regression modelling was used to determine factors associated with sexual or physical IPV, adjusting for age and survey design. About 34% of respondents had experienced IPV in the past year, with 21.4% reporting sexual and or physical forms. Past year experience of emotional and economic IPV were 24.6% and 7.4% respectively. Senior high school education or higher was protective of IPV (AOR = 0.51[0.30–0.86]). Depression (AOR = 1.06[1.04–1.08], disability (AOR = 2.30[1.57–3.35]), witnessing abuse of mother (AOR = 2.1.98[1.44–2.72]), experience of childhood sexual abuse (AOR = 1.46[1.07–1.99]), having had multiple sexual partners in past year (AOR = 2.60[1.49–4.53]), control by male partner (AOR = 1.03[1.00–1.06]), male partner alcohol use in past year (AOR = 2.65[2.12–3.31]) and male partner infidelity (AOR = 2.31[1.72–3.09]) were significantly associated with increased odds of past year physical or sexual IPV experience. Male perpetrated IPV remains a significant public health issue in Ghana. Evidence-based interventions targeting women’s mental health, disabilities, exposure to violence in childhood, risky sexual behavior and unequal power in relationships will be critical in reducing IPV in this setting.

## Background

Intimate partner violence (IPV) is an important global public health problem and contributes significant social and economic costs to societies [1, 2]. While both males and females could be victims of IPV, evidence shows a disproportionate prevalence among women [3]. IPV remains the most prevalent form of violence against women (VAW) worldwide; and global estimates of VAW suggest that 35% of all women will experience either IPV or non-partner sexual violence in their lifetime [4, 5].

IPV refers to any act of physical aggression, sexual coercion, psychological/emotional abuse or controlling behaviours by a current or former partner/spouse; and it includes any behavior within an intimate relationship that result in sexual, physical or psychologic harm [6, 7]. The immediate and later health consequences of IPV on victims include physical (death and injury), mental (depression, alcohol use problems), sexual and reproductive health risks (HIV, sexually transmitted infections, unwanted pregnancy and abortion and unfavorable pregnancy outcomes), and impaired social functioning [1, 3, 5].

Identified risk factors for IPV include history of violence in childhood, low education, alcohol and drug use, stress, communication challenges between partners, unequal power in relationships, unemployment status of male partners, gender inequitable masculinities and harmful attitudes to gender relations that result in female disempowerment and marginalization [8–14].

Based on data from a recent analysis by the WHO and London School of Hygiene and Tropical Medicine [4], the worst affected regions (based on countries with available data from various WHO regions) with respect to lifetime IPV experience are South-East Asia −37.7%, Eastern Mediterranean −37% and Africa −36.6%. Combining the prevalence of IPV and non-partner sexual violence shows a higher burden in Africa at 45.6% followed by South-East Asia −40.2%, Americas −36.1%, high income countries −32.7%, Western Pacific −27.9% and the least in Europe −27.2%.

The situation in Ghana is not very different from that reported for the entire African region. Coker-Appiah and Cusack reported that one in three Ghanaian women experienced physical abuse by male partners in their lifetime [15]. Reports from the 2008 Ghana Demographic and Health Survey (GDHS) indicated that 38.7% of ever married women surveyed had experienced any form of sexual, physical, emotional or all three forms of violence from a husband/partner in their lifetime. In the same 2008 GDHS report, past year experience of sexual, physical or emotional IPV was 34.9%; with about 20% experiencing physical or sexual forms in the past 12 months. Also, Ajah and Agbemafle reported that 33–37% of women had ever experienced abuse in an intimate relationship [16]. There is also evidence that IPV is prevalent among pregnant women in Ghana [17]. Recent findings from the Ghana Family Life and Health Survey indicate that violence is widespread among the Ghanaian population (15–60 years) with about 71% of both men and women surveyed having reported experience of at least one form of violence (both domestic and non-domestic) in their lifetime [10]. In this same report, 27.7% of women experienced at least one type of domestic violence in the last 12 months preceding the survey; with 23.3% experiencing two types of domestic violence.

The Government of Ghana in response to the calls for action on violence against women and girls (VAWG) by activists, NGOs and the global community has passed and/or amended several laws that protect the rights of women and girls. These include laws that provide for criminalizing of practices like female genital mutilation, widowhood rights, and discrimination based on sex and the Domestic Violence Act (Act 732) in 2007. Another action taken was the establishment of the institution of the Women and Juveniles Unit (WAJU) of the Ghana Police Service (currently named Domestic Violence and Victim Support Unit (DOVVSU)) in 1998 to deal with issues of domestic violence, which at the time was dominated by physical abuse by male spouses. In 2008, the National Policy and Plan of Work for various stakeholders to be involved in implementation of the Domestic Violence Act was developed under the supervision of the Ministry of Gender, Children and Social Protection (MoGCSP). However, the legislative instrument for the implementation of the act was only passed in 2016.

Following the publication of a nationwide study on VAWG in 1999, the Gender Studies and Human Rights Documentation Centre (Gender Centre) [18] developed and piloted a community based intervention known as the Rural Response System (RRS). Initial evaluation showed some positive effects on reduction of VAW in communities; and an impact evaluation of the RRS is currently underway in four districts of the Central Region of Ghana (registered on ClinicalTrials.gov Identifier: NCT03237585). This paper draws on the baseline assessment for the evaluation and presents the prevalence of IPV among ever-partnered women and the factors associated with past year experience of physical or sexual IPV. This paper compliments another which has been published on the prevalence and factors associated with male disclosed perpetration of IPV in the same study area [19].

## Methods

### Study population, tools and measures

This is a descriptive exploratory analysis conducted on the baseline survey of 2000 completed interviews (women only) of a two-arm unmatched cluster randomized control trial (RCT) assessing the impact of the Rural Response System’s (RRS) intervention. The Rural Response System (RRS) was designed as a community-based intervention to address major problems related to VAW in Ghana. These include poor institutional response to VAW, high degree of tolerance of VAW in the Ghanaian society due to strong perceptions that domestic violence is a private matter, the general confusion about what constitutes violence and ignorance about the causes, consequences and mechanisms that perpetuate VAW[15]. The RRS uses the strategy of trained community members known as Community-based action team (COMBAT) to undertake awareness-raising on gender-based violence as well as providing support to victims of violence to access justice. Additional details about the intervention design can be found on clinical trial.gov.

The RCT is being conducted in the Central Region of Ghana in 40 localities within four districts (two inland, two coastal). Both intervention and control districts have inland and coastal areas. Districts assigned to the control arm received no intervention while intervention districts received the RRS intervention. A list of localities (census enumeration areas-EAs) per district was obtained from the Ghana Statistical Service (GSS). A total of 40 localities (10 per district) were randomly selected from the list provided by simple balloting. Within each locality, different EAs were purposefully selected and designated male or female survey sites ensuring that they were separated as much as possible by space. Households were selected using multi-stage stratified cluster random sampling after households in each EA were listed based on EA maps obtained from the Ghana Statistical Service.

Females aged 18–49 years were interviewed for this survey. Randomly selected households based on a computerized software from the GSS, were visited and screened for eligibility. Eligible households had to have some adult female age 18–49 years, who had lived in the community for not less than 12 months preceding the survey, able to effectively communicate in either English, Twi or Fante languages, currently had or ever had a male partner, had no cognitive or speech challenges that affect ability to consent and had to be willing to participate. To ensure confidentiality and safety of respondents, only one eligible person was interviewed per household and simple balloting was employed in households that had more than one eligible female.

A structured quantitative survey tool was administered to respondents in face-to-face interviews with responses directly recorded onto a Personal Digital Assistant (PDA tablet). Questions covered general background and work characteristics of respondents, household food insecurity; economic situation and ease of accessing credit; life satisfaction and experience of childhood trauma. Additional questions assessed sexual behaviour, experience of IPV, prevailing social norms, ideas about gender relations and attitudes about relations between men and women. Questions relating to health and wellbeing, disability and substance use were also asked. The survey tool was adapted from the questionnaire used by the Stepping Stones and Creating Futures intervention study [20]. Questionnaire was pre-tested in a non-participating population similar to the survey communities in the Central Region for clarity, consistency and appropriateness of questions, expressions and response options. Appropriate adjustments were made to survey tool and tested prior to the main survey.

The main outcome for this paper is self-reported past year (12 months) experience of sexual or physical IPV. The measure was based on the WHO violence measure [21]. Questions on sexual (3-items) and physical (5-items) IPV experience were measured on a 4-point scale (1 = none, 2 = once, 3 = few, 4 = often) and an affirmative response to any of the 8-items (Table 1) qualifies one as a victim of sexual or physical IPV. Other types of IPV measured include emotional (4-items) and economic (1-item). Past year IPV was assessed based on details from respondents that were partnered in the past 12 months preceding the survey (n = 1877).

**Table 1:** List of items for measuring physical or sexual IPV.

In the last 12 months:
*Physical violence*
• How many times has your current or any previous husband or boyfriend slapped you or thrown something at you which could hurt?
• How many times has your current or any previous husband or boyfriend pushed or shoved you? • How many times has your current or any previous husband or boyfriend hit you with a fist or something else that could hurt?
• How many times has/did your current or any previous husband or boyfriend kick, drag, beat, choke or burnt you?
• How many times has your current or any previous husband or boyfriend threatened to use or actually used a gun, knife or other weapon against you?
*Sexual violence*
• How many times has a current or previous husband or boyfriend ever physically forced you to have sex when you did not want to?
• How many times has a current or previous husband or boyfriend, husband or partner used threats or intimidation (but not physical force) to get you to have sex when you did not want to?
• How many times has a current or previous husband or boyfriend ever forced you to do something else sexual that did not want to do?

Other covariates measured in this study include household food insecurity which was assessed using the abridged version of the Household Food Insecurity Access Scale (HFIAS) [22]. Questions covered 3 domains: anxiety and uncertainty about household food supply (e.g. “did you worry that your household would not get enough food”; insufficient quality (e.g. “did you or any household member have to eat a limited variety of foods due to a lack of resources”) and insufficient food intake and physical consequences (e.g. “did you or any household member go to sleep at night hungry because there was not enough food”). Households were categorized as severely food insecure, moderately food insecure, mildly food insecure and food secure. However, due to the relatively small numbers of respondents in the mild food insecure household groups, mild food insecure and food secure groups were collapsed into one group for analyses regarding household food insecurity.

Depression was assessed using the Centre for Epidemiological Studies Depression Scale (CES_D)[23]. Depression score was generated from all 20-items (Cronbach’s alpha = 0.86). Both past year and lifetime sexual behaviour was assessed using direct questions on number of main and multiple intimate partners, and 5 questions were used to measure engagement in transactional sex.

Childhood exposure to violence/ trauma was assessed using the Childhood Trauma Scale [24] which included 12 questions (Cronbach’s alpha = 0.73) covering neglect, witnessing of abuse of mother, sexual, physical, and emotional abuse. Childhood trauma was assessed on a continuous scale and binary outcomes were constructed for the sub-types of violence.

Substance use by both respondent and her current partner included direct questions on whether respondent or partner had used drugs or consumed alcohol in the last 12 months. Responses were categorized into whether the respondent or partner consumed alcohol in the past year preceding survey. Prevalence of substance use among both respondents and their male partners was very low hence all analyses were restricted to alcohol use.

Controlling behaviour of male partner was assessed using 8-items of the Gender Equitable Men’s (GEM) scale [25]. Items included: “When he wants sex he expects me to agree”; “If I asked him to use a condom, he would get angry”; “He won’t let me wear certain things”; “He has more to say than I do about important decisions that affect us”; “He tells me who I can spend time with”; “When I wear things to make me look beautiful he thinks I may be trying to attract other men”; “He wants to know where I am all of the time”; and “He lets me know I am not the only partner he could have”. An additive score of responses was generated based on a 4-point scale of 1-“strongly disagree” to 4-“strongly disagree”; with a higher total score representing higher control by male partner (Cronbach’s alpha = 0.70).

Individual attitudes and community gendered norms were measured using a 9-item gender relations scale adapted from Stepping Stones/Creating Futures Study in South Africa[20]. Internal consistency for both individual and community scales were 0.57 and 0.68 respectively. However, when 2- items, “I think that there is nothing a woman can do if her husband wants to have girlfriends” and “I think that if a man beats you it shows that he loves you” were dropped, the consistency for individual norms improved to 0.59. Thus, an additive score for individual norms was generated based on the 7-items. Also, two questions had to be dropped from the list of items measuring community norms to improve consistency, and these were: “My community thinks that if a wife does something wrong her husband has the right to punish her” and “My community thinks that if a man beats you it shows that he loves you”. A final additive score based on the 7-items (Cronbach’s alpha = 0.74) was used in analyses of community norms.

### Data analysis

A respondent was classified as having experienced IPV if they responded affirmatively to one or more of the questions relating to specific IPV forms (Table 1). Past year incidence of IPV was defined as a report of any IPV experience within the 12-months preceding the survey. Prevalence of lifetime experience of IPV was defined as the proportion of ever-partnered women who report any form of violence from an intimate partner at any point in their lifetime. Baseline prevalence of IPV, childhood trauma as well as other background characteristics of respondents with categorical measures are reported in proportions and 95% confidence intervals. Continuous variables including age of respondents, household food insecurity access score, relationship control score, depression score, disability score, number of biological children, number of sexual partners, and years lived in community are reported as means and standard deviations.

The main outcome for this paper is past year experience of sexual or physical IPV. Selected characteristics of respondents based on existing literature were described for women who had experienced sexual/physical IPV in past year or not. Independent samples t-test and chi-square tests were used to compare continuous and categorical characteristics of respondents respectively based on past year experience of sexual or physical IPV. Separate logistic regressions, adjusted for age and survey design, were run to examine the association between the main outcome variable and various aspects of respondent characteristics: background characteristics; gender norms; mental health, disability and substance use; childhood trauma; sexual behaviour and partner characteristics. A final multivariable logistic regression model was built using all variables tested at bivariate level, to determine the significant risk factors associated with past year sexual or physical IPV experience.

While the trial involved both male and female respondents, separate analyses were performed on baseline female survey (reported in this study) and that of males reported in our earlier study [19] to allow for a more in-depth examination of the prevalence and factors associated with male perpetration or female IPV experience in the study area. The separation of data analysis nevertheless, did not in any way compromise the statistical robustness of estimates reported in the two complementary works since relatively large sample sizes considered sufficient [26] were involved in this trial.

### Ethical considerations

This study obtained ethical clearance from the South African Medical Research Council Ethics Committee (Protocol ID # EC031-9/2015) and the Institutional Review Board of Noguchi Memorial Institute for Medical Research, University of Ghana (Protocol ID # 006/15-16). The trial protocol is registered on ClinicalTrials.gov (Identifier: NCT03237585). Prior to participation in the survey, the research assistants discussed the study’s participant information sheet and consent form with respondents. The discussion included adequate explanation of study objectives, potential risks, benefits, voluntary nature of participation, and confidentiality of information and trial procedures. All respondents provided written informed consent. Interviews were conducted in secluded places within or closest to respondent’s household to ensure privacy and safety of both interviewer and respondent. Respondents were assured of anonymity with the use of hand-held tablets for documentation of information and the use of unique identifiers that cannot easily be traced to them. Respondents were reimbursed with 10 Ghanaian Cedis (≈ 3 USD) for their time and inconvenience completing the questionnaire.

## Results

A total of 2000 adult female respondents aged between 18–49 years, with a mean (SD) age of 31.7 (8.6) years were surveyed at baseline for the community RCT. Over half (53%) of the respondents were married and about 16% were either divorced, separated or not in any heterosexual relationship at the time of interview. Sixty-three percent of our sample had worked or earned income in the past three months and less than a half of them reported no work or occasional work in the past year preceding the survey. Ninety percent of women had biological children with 3–4 children on average. Over 70% of respondents experienced household food insecurity with 37% experiencing severe food insecurity. Background details of respondents are shown in Table 2.

**Table 2:** Background characteristics of respondents (N = 2000).

			95% CI^¥^	
Characteristic	Frequency	% or mean	LCL	UCL
**Age of respondent (mean & CI)**		31.7	31.1	32.3
**Highest Educational level**				
None	434	21.7	18.3	25.6
Primary	459	23.0	20.7	25.4
Junior High school	897	44.9	40.8	49.0
Senior High school	156	7.8	5.9	10.2
Post-Secondary School	54	2.7	1.8	4.1
**Marital status**				
Married	1068	53.4	49.8	57.0
Divorced/separated/no relationship	268	13.4	11.3	15.8
Not married but in relationship	664	33.2	29.9	36.6
**Worked or earned Income in past 12 months**				
Each month	694	34.7	30.0	39.7
Most months	411	20.6	17.8	23.6
Once a while	504	25.2	21.8	28.9
Never Worked	391	19.6	16.1	23.5
**Worked or earned Income in past 3 months**	1253	62.7	55.9	69.1
**Household Food Security**				
Food Secure	448	22.4	19.9	25.1
Mildly insecure	103	5.2	3.7	7.2
Moderately insecure	704	35.2	31.8	38.7
Severely insecure	745	37.3	33.5	41.1
**Have Biological children**	1800	90.0	88.4	91.4
**Membership of groups**^§^	104	5.2	4.0	6.7
**Age at first marriage (mean & CI)**		22	21.5	22.4
**Years lived in community**^¤^ **(mean & CI)**		17.5	16.1	18.9
**Number of biological children (mean & CI)**		3.5	3.3	3.6
**Number of children financially supporting (mean & CI)**		3.6	3.4	3.7

^¥^ Estimation of the Confidence Interval took into account multi-stage design of the study.

^§^ Includes all social groups that require membership for association & participation.

^¤^ Number of years respondent has lived in the community where interview was collected.

Table 3 shows the lifetime and past year (12-month) experience of IPV among women interviewed. Half of women (50.9%) had experienced IPV in their lifetime; with ever experience of sexual or physical IPV (with or without emotional or economic IPV) being highly (39.3%) prevalent. A third had experienced physical violence (32.2%), a fifth (18.5%) sexual violence, 10.1% economic violence and 34.1% had experienced emotional violence in their lifetime. About 11% of women experienced both sexual and physical violence which could also include emotional violence in their lifetime.

**Table 3:** Prevalence of IPV experience among ever partnered women aged 18–49 years.

Characteristic	Past 12-month experience (N = 1877) ^§^				Lifetime experience (N = 2000) ^¤^			
			95% CI ^¥^				95% CI ^¥^	
	N	%	LCL	UCL	N	%	LCL	UCL
Sexual IPV^∞^	222	11.8	8.4	16.4	370	18.5	15.0	22.7
Physical IPV^∞^	290	15.5	12.7	18.7	643	32.2	28.3	36.3
Economic IPV^∞^	139	7.4	5.9	9.3	202	10.1	8.3	12.3
Emotional IPV	462	24.6	20.5	29.2	684	34.2	29.7	39.0
Both sexual and physical^∞^	110	5.9	3.9	8.7	228	11.4	9.1	14.2
Sexual and/or physical IPV^∞^	402	21.4	17.5	25.9	785	39.3	34.7	44.0
Any IPV^∞^	640	34.1	29.3	39.2	1019	50.9	46.0	55.9

^**§**^ Total number of women who had been in a relationship in the 12 months preceding the survey.

^¤^ Total number of women interviewed and who have ever been in an intimate relationship.

^¥^ Estimation of the Confidence Interval took into account multi-stage design of the study.

^∞^ Report for all IPV types may include emotional IPV.

**Fig 1:**
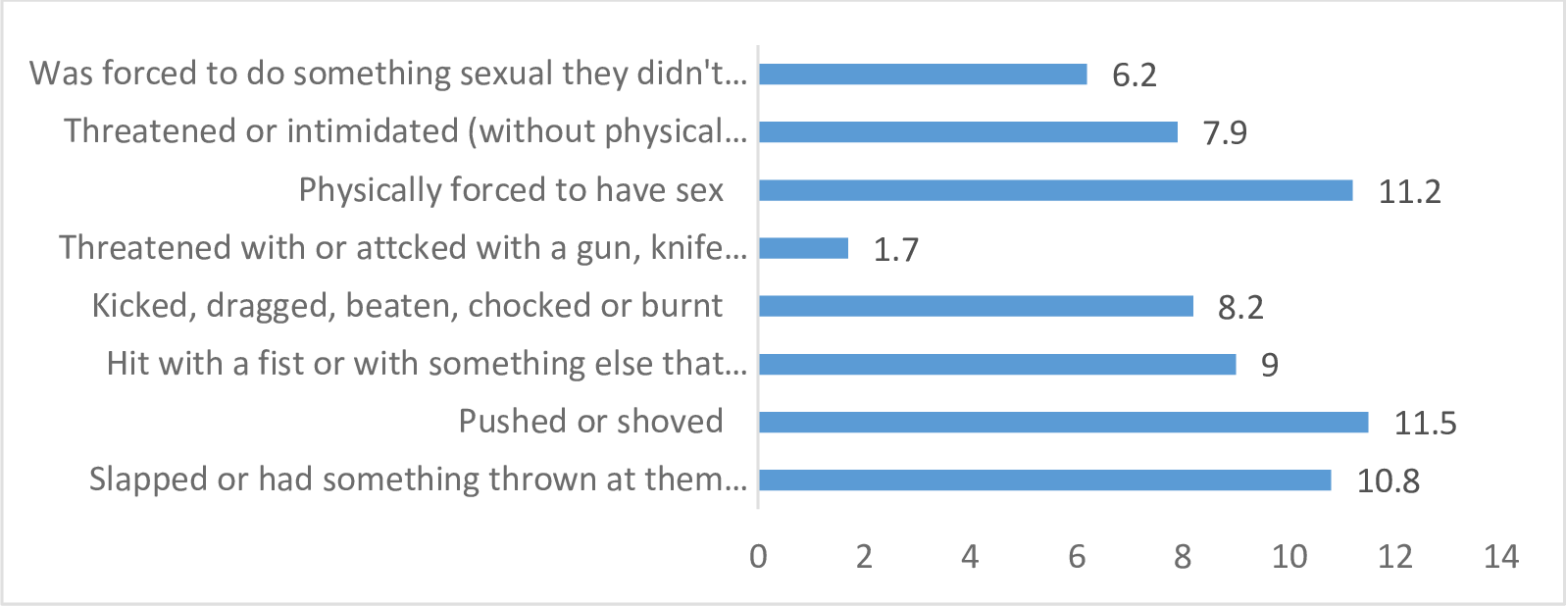
Prevalence of different forms of sexual or physical IPV experienced by women in past year.

Thirty-four percent of respondents had experienced IPV in the 12 months preceding survey which for many was sexual or physical in nature (21.4%). Prevalence of different forms of physical or sexual IPV experienced by women in the past year are shown in Fig 1. About a quarter (24.6%) of women had experienced emotional violence and 6% had experienced both sexual and physical IPV. Prevalence of economic violence in the past year was 7%.

Participant characteristics (background; gender attitudes and norms; mental health, substance use and disability; childhood trauma; sexual behaviour and partner characteristics) in relation to past year experience of sexual or physical IPV are shown in Table 4. Mean age of respondents, individual gender norms, community norms and life satisfaction were not different whether or not respondents experienced IPV in past year. However, women without experience of IPV in the past year had significantly better gender equitable attitudes compared to those with past year IPV experience (score of 14.8± 4.2 vs. 14.1± 3.9, p<0.05).

**Table 4:** Bivariate analysis of factors associated with past year sexual or Physical IPV experience.

			Sexual or Physical IPV Experience				
		No experience			Experienced		
Background Characteristics	N	n/mean^¤^		%/sd^¤^	n/mean^¤^	%/sd^¤^	p- value
**Respondent Age** ^ɤ^	1877	31.6		8.6	31.0	8.5	0.129
**Highest Educational level**							0.047
None	401	315		21.4	86	21.4	
Primary	426	325		22.0	101	25.1	
Junior High school	846	658		44.6	88	46.8	
Senior High school or higher	204	177		12.0	27	6.7	
**Marital status**							0.612
Married	1068	848		57.5	220	54.7	
Divorced/separated/no relationship	145	110		7.5	35	8.7	
Not married but in relationship	664	517		35.1	147	36.6	
**Household food security**							0.002
Food Secure + Mildly insecure	526	421		28.5	105	26.1	
Moderately insecure	666	546		37	123	29.9	
Severely insecure	685	508		34.4	177	44.4	
**Worked/earned in past 12mths**	1504	1168		79.2	336	83.6	0.076
**Gender attitudes and norms** ^ɤ^							
Gender attitudes (high = equitable)	1877	14.8		4.2	14.1	3.9	0.034
Individual norms (high = equitable)	1877	12.9		3.6	12.6	3.4	0.275
Community norms (high = equitable)	1877	11.5		4.5	11.0	4.3	0.143
**Mental Health, disability & alcohol use**							
Alcohol use in past year	102	61		4.2	41	5.4	<0.001
Depression score (high = depressed) ^ɤ^	1877	25.5		8.5	32.2	9.8	<0.001
Disability	140	76		5.2	64	15.9	<0.001
Life satisfaction ^ɤ^	1877	14.7		4.0	14.9	4.0	0.296
**Childhood trauma**							
Witnessed abuse of mother	276	167		11.3	109	27.1	<0.001
Experienced childhood physical abuse	709	511		34.6	198	49.3	<0.001
Experienced childhood sexual abuse	507	338		22.9	169	42.0	<0.001
Experienced childhood emotional abuse	820	608		41.2	212	52.7	<0.001
Was neglected in childhood	1206	897		60.8	309	76.9	<0.001
**Sexual behavior**							
Multiple sexual partners in past year	115	48		3.3	67	16.8	<0.001
Transactional sex in past year	142	68		4.7	74	18.6	<0.001
**Partner Characteristics**							
Controlling behaviour (high = controlling) ^ɤ^	1877	19.9		5.1	21.2	5.2	<0.001
Alcohol or drug use	446	289		19.5	157	39.4	<0.001
Alcohol use in past year	434	278		18.7	156	39.0	<0.001
Not confident in partner fidelity	485	303		20.5	182	45.3	<0.001

Summary statistics represented by n/mean and %/standard deviation.

^ɤ^ p-values are from simple regression analysis; all other p-values are from chi-square tests.

Significantly higher proportions of respondents who reported some form of disability (15.9% vs. 5.2%, p<0.001), higher depression score (32.3± 9.8 vs. 25.5 ± 8.5, p<0.001), higher male partner control score (21.2 ± 5.2 vs. 19.9 ± 5.1, p<0.001), reported alcohol use by male partner (39.0% vs. 19.5%, p<0.001) and a lack of confidence in partner fidelity (45.3% vs. 20.5%, p<0.001) experienced IPV in the past year compared to respondents who did not have those characteristics (see Table 4). Significantly more respondents who experienced past year IPV had severe household food insecurity compared to those who had no IPV experience (44.4% vs. 34.4%, p<0.01). Work in past year did not differ among respondents based on past year IPV experience at the bivariate level of analysis.

A significant proportion (64.3%) of respondents reported having been neglected as children; with a higher proportion of them having experienced IPV in past year compared to those who did not experience neglect (76.9% vs. 60.8%, p<0.001). More respondents who experienced past year IPV reported experience of childhood emotional, physical and sexual abuse compared to those with no childhood experience of trauma (52.7% vs. 41.2%; 43.3% vs. 34.6%; 42.0% vs. 22.9%, all p<0.001; respectively).

Regarding sexual behaviour, significantly more respondents who experienced past year IPV had multiple sexual partners (16.8% vs. 3.3%, p<0.001) or engaged in transactional sex (18.6% vs. 4.7%, p<0.001) compared with those with no experience of IPV in past year. Women’s alcohol use in past year was more prevalent among respondents who experienced IPV compared to those who did not experience IPV in past year (5.4% vs. 4.2%, p<0.001).

Table 5 shows the final model of factors associated with past year experience of sexual or physical IPV among respondents. Increasing age of respondents was protective of past year IPV experience (AOR = 0.96, 95% CI = 0.94–0.98, p = 0.001). Senior secondary school education or higher reduced the odds of past year IPV experience by 49% (p<0.05) compared to having no formal education. Respondents from moderately food insecure households had reduced odds of past year IPV experience (AOR = 0.68, 95%CI = 0.46–0.98, p<0.05) compared to their counterparts who were food secure or mildly food insecure. Each increase in severity of depression was associated with a 6% increase in odds of past year IPV experience. Having disability was associated with over two times the odds of experiencing past year sexual or physical IPV compared to persons without disability (AOR = 2.30, 95% CI = 1.57–3.35, p<0.001).

**Table 5:** Multivariate analysis of factors associated with past year sexual or Physical IPV experience.

	Unadjusted OR	95%CI^¥^	Adjusted OR	95%CI	p-value^§^
**Background Characteristics**					
**Respondent Age**	0.98	0.96–1.00	0.96	0.94–0.98	**0.001**
**Highest Educational level**					
None	—				
Primary	1.10	0.72–1.68	0.85	0.54–1.34	0.470
Junior High school	0.99	0.67–1.47	0.91	0.57–1.46	0.701
Senior High school or higher	0.56	0.40–0.78**	0.51	0.30–0.86	**0.014**
**Marital status**					
Married	—				
Divorced/separated/no relationship	1.23	0.82–1.86	0.67	0.41–1.08	0.099
Not married but in relationship	1.07	0.74–1.55	0.94	0.64–1.38	0.742
**Household food security**					
Food Secure + Mildly insecure	—				
Moderately insecure	0.86	0.62–1.18	0.68	0.46–0.98	**0.041**
Severely insecure	1.34	1.04–1.72*	0.83	0.59–1.17	0.277
**Worked/earned in past 12mths**	1.33	0.94–1.88	1.45	1.04–2.04	**0.030**
**Gender attitudes and norms**					
Gender attitudes (high = equitable)	0.96	0.92–1.01	0.99	0.95–1.04	0.817
Individual norms (high = equitable)	1.02	0.97–1.06	1.04	0.98–1.11	0.178
Community norms (high = equitable)	0.99	0.95–1.03	1.00	0.96–1.04	0.904
**Mental Health, disability & alcohol use**					
Alcohol use in past year	2.13	1.25–3.61**	1.36	0.76–2.43	0.285
Depression score (high = depressed)	1.08	1.06–1.10***	1.06	1.04–1.08	**<0.001**
Disability	2.91	2.03–4.15***	2.30	1.57–3.35	**<0.001**
Life satisfaction	0.98	0.95–1.00	0.99	0.96–1.02	0.502
**Childhood trauma**					
Witnessed abuse of mother	2.32	1.81–2.99***	1.98	1.44–2.72	**<0.001**
Experienced childhood physical abuse	1.49	1.14–1.94**	1.28	0.93–1.76	0.121
Experienced childhood sexual abuse	1.91	1.50–2.42***	1.46	1.07–1.99	**0.019**
Experienced childhood emotional abuse	1.07	0.83–1.38	0.95	0.74–1.22	0.680
Was neglected in childhood	1.64	1.26–2.14**	1.19	0.85–1.68	0.296
**Sexual behavior**					
Multiple sexual partners in past year	3.60	2.16–6.01***	2.60	1.49–4.53	**0.001**
Transactional sex in past year	2.74	1.92–3.92***	1.73	1.15–2.62	**0.011**
**Partner Characteristics**					
Controlling behaviour (high = controlling)	1.04	1.02–1.07**	1.03	1.00–1.06	**0.033**
Alcohol use in past year	2.78	2.13–3.63***	2.65	2.12–3.31	<0.001
Not confident in partner fidelity	2.87	2.25–3.66***	2.31	1.72–3.09	**<0.001**

^§^ P-values shown are for multiple regression analyses.

^¥^ Bivariate associations [OR] significant at

* = p<0.05,

**p<0.01,

***p<0.001.

Women who witnessed abuse of their mother in childhood had almost twice the odds of experiencing past year IPV (AOR = 1.98, 95%CI = 1.44–2.72, p<0.001), while experiencing childhood sexual abuse was associated with a 46% increase in odds of past year IPV experience (AOR = 1.46, 95%CI = 1.07–1.99, p<0.05). Having multiple sexual partners in past year predicted about two-and-a-half-fold higher odds of experiencing IPV (OR = 2.60, 95%CI = 1.49–4.53, p = 0.001) while engaging in transactional sex predicted 73% increased odds of experiencing IPV (OR = 1.73, 95%CI = 1.15–2.62, p<0.05). Every unit increase in score for controlling behaviour of the male partner was associated with a 3% increase in odds of past year physical or sexual IPV experience. Alcohol use by male partner was associated with over two-and-a-half-fold increase in odds (AOR = 2.65[2.12–3.31]) of women experiencing physical or sexual IPV in past year. Also, a lack of confidence in male partner fidelity was associated with over two times the odds of experiencing IPV in past year (OR = 2.33, 95%CI = 1.75–3.12, p<0.001).

## Discussion

The prevalence of past year intimate partner violence (IPV) experience (physical, sexual, emotional) recorded in this study is very similar to the findings reported by 2008 Ghana Demographic and Health Survey [27]. Our reports of past year experience of sexual or physical IPV were much higher than that estimated by the most recent report on domestic violence in Ghana [10]. This increased prevalence is likely due to two reasons. Firstly, the Ghana Family Life and Health Survey (GFLHS 2015) reported estimates of ‘domestic violence’ which may not necessarily have been perpetrated by intimate male partners, who are the most likely perpetrators of sexual and physical violence against women. Secondly, our study was limited to ever partnered women (18–49 years) and not a wider female population (15–60 years) as sampled by the GFLHS 2015.

Our study population had similar demographic characteristics to the general Ghanaian women population as reported by the 2014 GDHS [28]. However, an unusually high proportion of our respondents (21.7%) had no formal education, compared to estimates for the Central Region (5.1%) or general rural populations in Ghana (15.3%). This lack of formal education indicates low female literacy and empowerment which has direct impacts on IPV risk and overall well-being [29]. Formal education improves access to knowledge and information necessary in making demands for social change geared towards more equitable standards of living for women. Our finding on the association between educational attainment and risk for IPV experience is consistent with general findings from the WHO multi-country study on women’s health and domestic violence amongst others [27, 30, 31] which found that attaining a senior secondary school education or higher was protective of IPV experience. This relationship may stem from the fact that educated women are more likely to be exposed to information necessary for better management of interpersonal relationships as evidenced in Malawi [32]. Similarly, an examination of 2003 and 2008 DHS data on Ghana revealed that women who had attained senior high school education or higher were less likely to approve domestic violence against women compared to women with lower or no education [33]. Furthermore, women’s educational attainment is closely linked with increased probability of having paid jobs, reduced odds of status loss and a better balance of power in marriage [34, 35], which can grossly reduce economic dependency, a phenomenon known to increase risk of IPV among Ghanaian women (31). Evidence from the 2008 GDHS showed that women who were employed but not for monetary gain were more likely to experience physical, sexual and emotional violence [27]. Recent national data showed that women in the Central region were more likely than women in other parts of Ghana to own land/property, and earn same as or more than their husbands [28]. Generally, women’s ownership of assets and higher education have been found to be protective of IPV risk [36]. Although recent (2014) DHS data show a slight reduction in percentage of Ghanaian women who are employed (91% in 2008 vs. 87% in 2014), the majority (65%) of those who earn cash independently decide on how to use their money [28]. The DHS evidence further shows increased proportion of women involved in decision making regarding their health, household purchases and visit to family. This improvement in women’s empowerment is positively associated with educational attainment of women; and the benefits of education thus, cannot be overemphasized in this population. Nevertheless, some findings among similar populations suggest a potentially U-shaped relationship between women’s educational attainment and IPV risk [11, 30]. Similarly, a review by Vyas and Watts [36] reported mixed findings regarding relationship between women’s engagement in income generating activities and IPV risk. Although evidence from the WHO multi-country study on women's health and domestic violence [30] found no association between IPV experience and employment status, our data indicated higher odds of past year IPV experience among women who were employed. This has implications for IPV prevention in Ghana considering that it has a relatively high proportion of women in paid employment. A research to examine how IPV risk is independently influenced by either women’s empowerment and asset ownership or women’s employment or both in this setting would be invaluable. Effective IPV prevention programmes therefore, will need to sustain achievements in female empowerment while improving gender empowerment and skills training [37] to address predisposing factors that heighten women’s IPV risk in general and especially with employment.

IPV risk is known to be higher in households that are economically disadvantaged or under economic stresses [38–41]. Food insecurity itself is a type of economic hardship that results in a lack of adequate food for members of the household and can cause mental distress [42–48]. Our study found a weak and inconsistent association between IPV experience and household food insecurity; with moderate food insecurity being associated with reduced odds of past year IPV experience. Our findings are contrary to evidence from California that showed a strong association between food insecurity and IPV experience with higher odds of IPV observed among those with worsening food insecurity [49]. This discrepancy in our findings deviates from the multitude of literature linking food insecurity and IPV risk [41, 44, 47, 50–52], suggesting some level of confounding between these two variables in our population. Further examination and modelling of these associations in our population is therefore warranted.

Our study found a positive association between increasing severity of depression and IPV risk. IPV experience has negative consequences on mental health of victims [1, 53]. The relationship between IPV risk and onset of depressive symptoms are bidirectional. A longitudinal study by Ouellet-Morin and colleagues showed that women who experienced IPV had two-fold risk of suffering a new onset depression (49). Whether IPV precedes depression or vice-versa, mental health symptoms present significant challenges to how victims manage, function and cope with the ‘violent’ environment [53, 54] which can further worsen incident IPV. In addition, recurrent IPV victimization is associated with worsening mental health [55]. There is some evidence to suggest that the link between food insecurity and IPV risk among women is mediated by depression [51, 56]. Depression among women likely increases feelings of helplessness, and a lack of both motivation and ability to look for food, worsening the odds of hunger in the household [38].

Depression among IPV victims has also been linked with observing IPV and or experiencing violence in childhood [55, 57]. Our study found an association between IPV experience and witnessing of abuse of mother in childhood as well as experiencing childhood sexual abuse. These observations are consistent with evidence from the WHO multi-country study on women’s health and DV [30]. Aside from the direct link between experience of depression or other mental health morbidities following childhood abuse and IPV, other intermediary factors include risky adolescent and adult behaviour that increase vulnerability to IPV victimization and re-victimization [58]. While our study did not examine the relationship between childhood exposure to violence and current sexual behaviour, IPV risk was significantly increased for women who engaged in transactional sex and or had multiple sexual partners (had sex with other(s) who were not main partner(s)) in the past year. Our finding of increased IPV risk with having multiple sexual partners in past year can be explained by the reported use of physical violence by men to control or punish women who have many partners [59]. There is evidence that the use of physical violence against women with many partners can even be under mere suspicion of infidelity by a husband or partner [60].

Our findings also show high prevalence of childhood trauma including neglect, which were all associated with increased odds of IPV risk at bivariate level. However, witnessing abuse of their mother remained a significant predictor of increased IPV risk as documented elsewhere among both men and women [9, 11, 30]. Childhood exposure to IPV in the home increases the chances of normalization, acceptance or internalization of IPV as normal or inevitable [61, 62]. The acceptance of violence or approval of domestic VAW have been found to be associated with increased IPV risk [3, 63]. Generally, in Ghana, there is a reduction in proportion of women who approve the use of physical violence against wives by husband (37%-2008 GDHS; 28%- 2014 GDHS), although approval of physical violence was higher among rural, less educated, poorer and married women [27, 28].

Our study found that an increase in either types or severity of disability among women was associated with increased odds of past year IPV experience. Women with disabilities typically present with increased risk of IPV compared to those without disabilities [64–66]. Persons with disability are more likely to be poorer, have less education, be physically dependent on intimate partner and be perceived as vulnerable by the partner [67].

Our study showed increased odds of experiencing IPV with increasing control exerted by male partner/husband; and this finding is consistent with the 2008 GDHS data [27]. Evidence from the GDHS suggests that more men are increasingly embracing female equality and empowerment as evidenced by decision making, control of resources and allowing women’s paid job [27, 28]. However, they still may hold strict views about gender [68] and any breech of defined roles could result in violence [61, 64]. In most patriarchal societies including Ghana, males are regarded to be superior to the females, have more power and voice in intimate relations and expected to ‘control and discipline’ their women and households [69]. Our study found significantly higher IPV experience among women reporting higher control by partner and those who lacked confidence in their partner’s fidelity. Male control and infidelity are very much related in this context where male superiority is expressed through ‘control of women and sexual prowess [68]. The payment of bride price across ethnic groups in Ghana often further entrenches the male superiority and entitlement to sex, which can also result in marital rape for non-consenting wives [70, 71]. While the proportion of women accepting the use of physical violence by husbands against wives is gradually reducing in Ghana [28, 33], the complexity of gender relations in patriarchal societies such as Ghana cannot be underestimated [10, 11, 31, 61, 64]. Male partner alcohol use was a significant risk factor for women’s past year IPV experience in this study was alcohol use; a finding consistent with other studies [72–74]. The role alcohol plays in IPV perpetration is both of direct physiological disinhibitory effect or peoples’ expectation of its disinhibitory effect [74], often resulting in violent behaviour.

Prevalence of past year sexual or physical IPV experience reported in this study is comparable to estimates of male reported perpetration of sexual or physical IPV perpetration reported in an earlier complementary study among men in the same trial [19]. Both male and female data showed childhood exposure to violence, risky sexual behaviour in both men and women and male substance use as significant risk factors for past year experience and perpetration of physical or sexual IPV in the study area. Our earlier study [19] also reported gender inequitable norms and female partner unemployment to be significantly associated with increased odds of male IPV perpetration, which was not supported by data on women’s IPV experience in the same study area. The lack of association between past year physical or sexual IPV experience by women and gender inequitable norms was due to the generally homogenous and high gender inequitable norms recorded among women in the study area. On the contrary, women’s survey data showed higher odds of IPV experience among employed women compared to their unemployed counterparts. The dynamics by which female employment and unemployment leads to both an increased risk for IPV experience and reported perpetration of IPV among women and men respectively warrants future investigation in the study area.

The findings of this baseline assessment of a RCT assessing intimate partner violence (IPV) in four districts of the Central region of Ghana presents some limitations that must be mentioned. Firstly, this was a cross-sectional survey and causality cannot be assumed based on associations between participant characteristics and past year IPV experience. Secondly IPV experience may have been underreported in this population; similar to other settings, due to social desirability or fear of further abuse or stigmatization that could result from reporting or the culture of silence [75]. In order to minimize this risk, this study fully complied with ethical and safety recommendations for research on domestic violence against women [76]. Every effort was made to reduce distress to respondents by: asking questions in a supportive and non-judgmental manner; interviewers were trained on how to terminate interviews when impact of questions was perceived as negative by the respondent; referral of respondents requiring assistance to a social worker and assuring respondents of confidentiality of responses. Nevertheless, the wide range of standard factors measured by the study allows for comparison across different settings and provide insights to understanding IPV risk in this setting.

## Conclusion

One in three women experienced some form of IPV in the past year preceding the survey and about 22% of women experienced physical or sexual IPV. Factors significantly associated with physical or sexual IPV risk among women were education, paid employment, depression, disability, exposure to violence in childhood, multiple sexual partners and male partner control, alcohol use and infidelity. Although advances have been made regarding women’s empowerment through education and legal structures in Ghana, there is the need to: (1) develop evidence-based culturally-appropriate interventions that address unequal power relations between men and women, making women and girls more vulnerable to IPV; (2) enforce child protection laws and laws protecting the rights of persons with disability; (3) design appropriate education materials for improving communication skills of women and intimate couples; and (4) strengthen state institutions to better manage victims of IPV and child abuse.

## Supporting information

S1 Data (CSV)
